# Spitting Blood

**Published:** 1884-03

**Authors:** 


Spitting blood is present in perhaps two-thirds of all who die of consumption.

When blood appears as a mere speck or drop or streak in the saliva, it is not a symptom worthy of notice. In any other form, it is the knell of death in men. In women, the mere spitting of blood, if during the periods, is no critical symptom; does not indicate the presence of tubercles necessarily. In men, it does. That is, when the blood is mixed up with the saliva, or comes clear, from half a teaspoonful at a time to a quart, tubercles are largely present, and in about two years the man will die, unless this symptom is removed.

Spitting of blood relieves the over fullness of the lungs, and diminishes cough remarkably, sometimes. Thus it is that bringing up a mouthful or two at a time, at intervals, is a relief, and may protract life for several years longer than would have been the case had it not been a symptom. Women losing blood naturally and periodically, thus protract the disease indefinitely

We have recorded the birth of tubercles as founded in a want of sufficient distension of the air-cells by full breathing, to give the blood-tubes a direct line of conveyance.

But it is important to observe that precisely the same result will follow, if the blood becomes thick, mush-like. The blood-tubes may be ever so straight, yet if the blood be thick with impurities or from being of an imperfect material, the blood vessels will, if but moderately distended, allow the oozing through of its thinner particles, and give rise to tubercle. If the distention is intensified, then they burst their sides, and there is *Hœmorrhage of the Lungs.* In plain English, spitting of blood.

Thus we have come to two great important practical facts:

The want of full breathing gives birth to tubercle.

The want of pure blood gives birth to tubercle.

And here we have the two universal causes of Consumption:

